# A New Synthetic Route to Polyhydrogenated Pyrrolo[3,4-*b*]pyrroles by the Domino Reaction of 3-Bromopyrrole-2,5-Diones with Aminocrotonic Acid Esters

**DOI:** 10.3390/molecules22112035

**Published:** 2017-11-22

**Authors:** Khidmet Shikhaliev, Artem Sabynin, Valeri Sekirin, Michael Krysin, Fedor Zubkov, Kristina Yankina

**Affiliations:** 1 Department of Organic Chemistry, Faculty of Chemistry, Voronezh State University, 1 Universitetskaya sq., Voronezh 394018, Russia; chocd261@chem.vsu.ru (K.S.); artemsabynin@rambler.ru (A.S.); sekirin.valeriy@mail.ru (V.S.); 2 Department of Organic Chemistry, Faculty of Physics and Mathematics and Natural Sciences, RUDN University, 6 Miklukho-Maklaya St., Moscow 117198, Russia; fzubkov@sci.pfu.edu.ru (F.Z.); aleksandrina21@mail.ru (K.Y.)

**Keywords:** pyrrole, pyrrolo[3,4-*b*]pyrrole, bromomaleimide, aminocrotonate, domino reaction

## Abstract

A new synthetic approach to polyfunctional hexahydropyrrolo[3,4-*b*]pyrroles was developed based on cyclization of N-arylbromomaleimides with aminocrotonic acid esters. A highly chemo- and stereoselective reaction is a Hantzsch-type domino process, involving the steps of initial nucleophilic C-addition or substitution and subsequent intramolecular nucleophilic addition without recyclyzation of imide cycle.

## 1. Introduction

Bicyclic pyrrolopyrroles are the core of numerous compounds with various useful properties. For example, they are used as optoelectronic materials [1,2], pigments for varied purposes [3,4,5,6], and are characterized by a variety of biological activities [7,8]. Inhibitors of protein methyltransferases [9], glycosyltransferases [10], agonists of various serotonin 5-HT-receptors [11,12,13], antagonists of integrin VLA-4 [14], and promising structural analogs of antibacterial fluoroquinolones [15] have been found among the derivatives of hydrogenated pyrrolo[3,4-*b*]pyrroles, thereby causing a significant interest in the search for new synthetic approaches to this heterocyclic system.

**Scheme 1:**
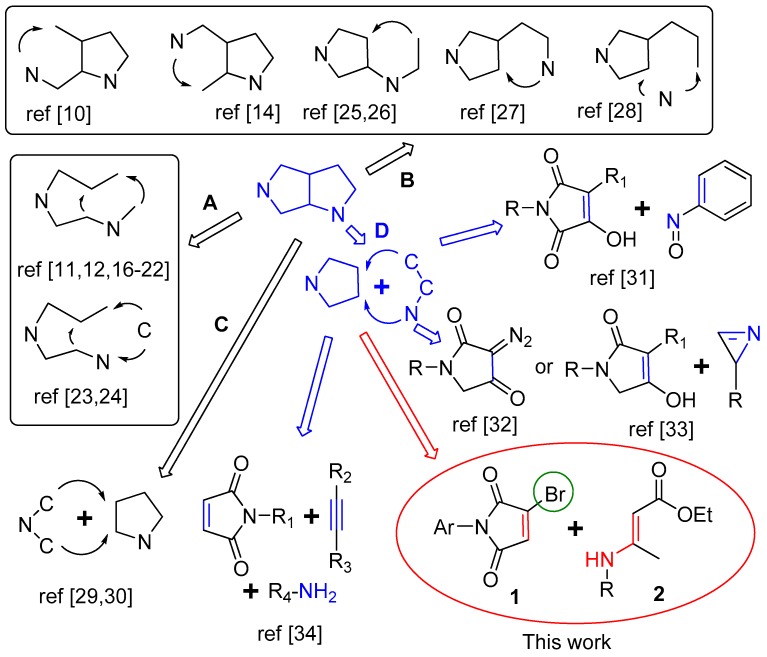
Retrosynthetic routes to pyrrolo[3,4-*b*]pyrroles.

The most common strategy for its construction from non-cyclic precursors (Scheme 1, Route A) is based on a multistage synthesis of N-alkenyl tethered aldehydes, which are further subjected to intramolecular cyclization with α-amino acids via the formation of azomethine ylides [11,12,16,17,18,19,20,21]. Hydrogenated pyrrolo[3,4-*b*]pyrroles are also formed as a result of intramolecular 1,3-dipolar cycloaddition in phosphorus-containing azomethine ylides generated in situ from alkene-tethered imines, acid chlorides and phosphonites [22], or transition metal promoted carbonilative [2+2+1] carbocyclization of N-allene imines [23,24].

Another direction in the synthesis of pyrrolo[3,4-*b*]pyrroles is the anellation of the second pyrrole ring. Examples of such reactions include intramolecular amidation [10,14], alkylation [25], condensation [26], nucleophilic addition [27], and Paal–Knorr heterocyclization [28] in appropriately substituted pyrroles (Scheme 1, Route B). Intermolecular convergent approaches to this heterocyclic system have been realized to a much lesser extent. Thus, the dipolarophilic pyrrole derivatives are anellated over the *b* bond as a result of the 1,3-dipolar cycloaddition of azomethine ylides, generated from silylated hemiaminals [29] or aziridines [30] (Scheme 1, Route C). Hexahydropyrrolo[3,4-*b*]indoles were obtained as a result of organocatalytic asymmetric anellation of N-hydroxymaleimides with nitrosobenzene [31]. Synthetic equivalents of C–C–N synthon are also 2*H*-azirines, which form hexahydropyrrolo[3,4-*b*]pyrroles in Cu-catalyzed domino-reactions with tetramic acid derivatives [32,33]. Oxidative cyclization of maleimides with amines and alkyne esters is also catalyzed by copper (I) salts [34] (Scheme 1, Route D). It should be noted that almost all of these reagents are not readily available.

In continuation of our research on the synthesis of heterocycles based on cyclic imides of unsaturated dicarboxylic acids [35,36], in the present work we report the unusual domino reaction of 1-aryl-3-bromo-1*H*-pyrrole-2,5-diones (bromomaleimides) (**1**) with N-substituted esters of β-aminocrotonic acids (**2**) for the metal-free preparation of a series of new hexahydropyrrolo[3,4-*b*]pyrroles.

## 2. Results and Discussion

**Scheme 2:**
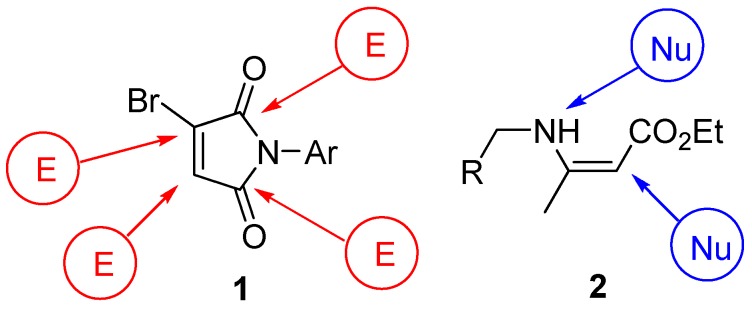
Reaction centers in bromomaleimides (**1**) and aminocrotonates (**2**).

One of the most interesting applications of maleimides, which do not have substituents on the C=C bond, in the synthesis of heterocyclic compounds are domino-recyclization reactions with a variety of dinucleophilic reagents [37,38,39]. Despite the presence of three electrophilic centers in their structure (one of the double bond carbon atoms and two carbonyl C atoms), a fairly high regioselectivity was noted for similar reactions, in particular, with aminocrotonic acid esters as 1,3-C,N-dinucleophiles [40]. Our choice of 1-aryl-3-bromo-1*H*-pyrrole-2,5-diones (**1a**–**d**) is due, on the one hand, to their easy synthetic availability [41] and, on the other hand, the appearance of yet another, compared to the C-unsubstituted maleimides, electrophilic C-atom, to which a bromine atom is bound, which significantly expands the variety of possible transformations. N-substituted ethyl aminocrotonates (**2a**–**c**), also easily synthesized by known methods [42,43], were chosen for the purpose of structural diversification of the target substances (Scheme 2).

**Scheme 3:**
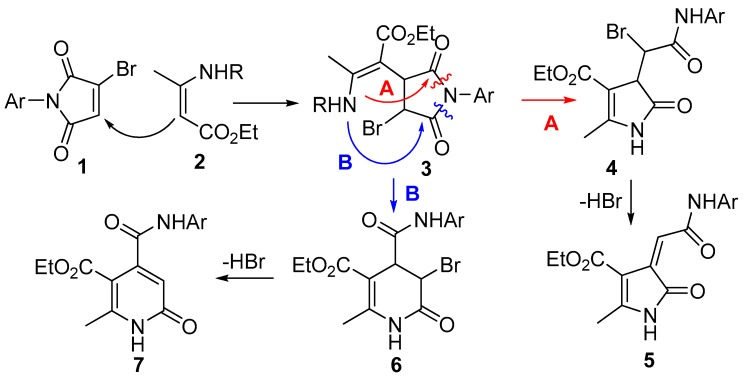
Probable direction of reclyzation of **1**.

According to the well-known literature data on the reactions of maleimides with 1,3-C,N-dinucleophiles [35,37,40], we assumed that the most probable direction of interaction of bromomaleimides (**1**) with aminocrotonates (**2**) will be a Michael-type reaction, followed by intramolecular transamidation with simultaneous recyclization of the imide cycle in an intermediate (**3**). Depending on the carbonyl atom at which the last reaction takes place, either dihydropyrroles (**4**) or tetrahydropyridines (**6**) can form. Their dehydrobromination can lead to pyrrolinone (**5**) or pyridinone (**7**) (Scheme 3).

**Scheme 4:**
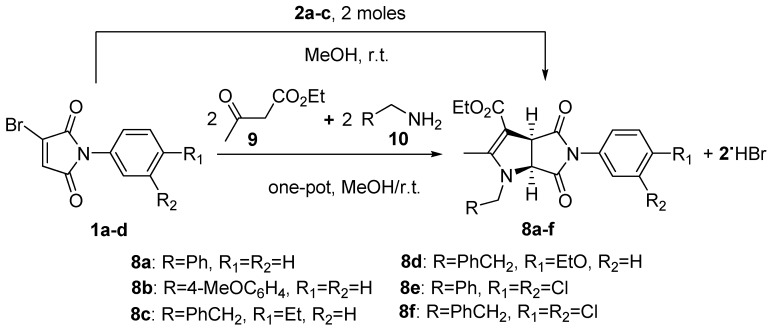
Reaction between **1** and **2**.

Monitoring of the reaction conditions for the example of **1a** and **2a** by TLC showed that, for their reactions, stirring in methanol without heating for 5 h is sufficient. Similar results were obtained in acetic acid, but the product yield was lower. In other solvents (chloroform, ethyl acetate, benzene, and dioxane), either reagent conversion was insignificant under the given conditions or a complex mixture of substances was observed to form when heated. In a **1a**/**2a** molar ratio of 1:1, part of the starting bromomaleimide does not react, while the aminocrotonate reacts completely. Total conversion of bromomaleimide is achieved with a molar ratio of reactants of 1:2. The second molecule of aminocrotonate probably binds the hydrogen bromide liberated during the reaction. The reaction at a reactant molar ratio of 1:1 and the same amount of the additional base (Et_3_N or pyridine) resulted in the formation of tar products with a significant decrease in the yield of the target substances. Thus, only one product was formed: (3*aS*,6a*R*)-ethyl 1-benzyl-2-methyl-4,6-dioxo-5-phenyl-1,3*a*,4,5,6,6a-hexahydropyrrolo[3,4-*b*]pyrrole-3-carboxylate (**8a**) instead of the expected **4**–**7** (Scheme 4).

Considering the simplicity of obtaining **2**, we attempted multicomponent one-pot synthesis of pyrrolopyrroles (**8**). Preliminarily, the mixture of equimolar amounts of ethyl acetoacetate (**9**) and appropriate amine (**10**) was stirred for 24 h, after which, without isolation of the resulting aminocrotonate, a methanol solution of half the amount of corresponding brommaleimide was added to the solution. Stirring was continued for 4 to 6 h (TLC control). The yields of the target substances isolated by simple filtration proved to be comparable with the two-component variant (Table 1).

In the ^1^H NMR spectra of pyrrolopyrroles (**8a**–**f**), in addition to the well identifiable signals of the substituents, there are a doublet of doublets or broadened doublet of H-6a (about 4.30 ppm with the vicinal spin–spin coupling constants J_H-3a-H-6a_ ~10.5 Hz and the long W-constant ^4^J_H-C-N_^1^_-C-H_^6a^ ~1.0 Hz), as well as the doublet H-3a at ~4.50 ppm for N^1^-benzyl derivatives **8a,b,e** and ~4.75 ppm for N^1^-phenethyl derivatives **8c,d,f** (J_H-3a-H-6a_ ~10.5 Hz). It is the multiplicity of the proton signal H-6a that proves its location. Diastereotopic are methylene protons, which are part of the benzyl, ester and phenethyl groups, causing the last two groups of complex type of appropriate signals. The absence of NH-signals excludes the formation of alternative compounds **4**–**7** (Scheme 3).

**Figure 1:**
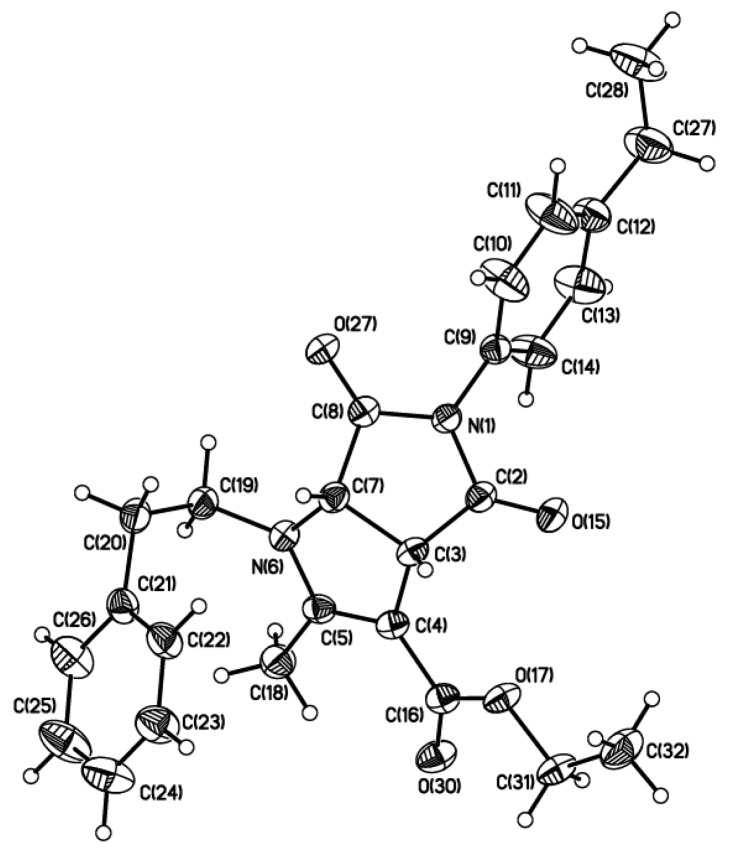
Molecular structure of pyrrolopyrrole (**8c**).

Conclusions on the arrangement of the substituents, based on ^1^H NMR data, are confirmed by the results of X-ray analysis of **8c** (Figure 1; non-hydrogen atoms are represented by probabilistic ellipsoids of atomic displacements (*p* = 0.5)). Thus, the interaction of **1** with **2** proceeds chemo- and stereoselectively without recycling of the imide cycle and leads to the formation of hexahydropyrrolopyrroles (**8**) with two adjacent quaternary asymmetric centers in their structure.

**Scheme 5:**
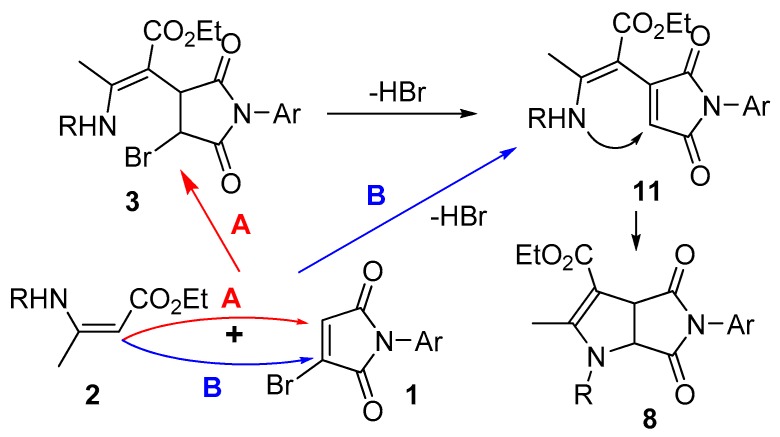
Possible sequences of reactions in the cascade formation of pyrrolopyrroles (**8**).

The polyelectrophilic character of **1** and the dinucleophilic character of **2a** (Scheme 2) cause a variety of possible variants of the initial interaction of the reagents and the direction of further transformations, both with opening and without opening the imide cycle. In our opinion, only two reasonable synthetic schemes can lead to the formation of **8a**–**f**: (a) a Michael-type nucleophilic C-addition of amino crotonate at the C-4 maleimide atom followed by dehydrobromination of succinimide (**3**) and a subsequent intramolecular cyclization of the intermediate **11** as a result nucleophilic addition with the participation of the nitrogen atom of the enamine fragment (Path A); or (b) direct nucleophilic substitution of the bromine atom in the imide **1**, also involving the C atom of the aminocrotonate and the subsequent analogous cyclization (Path B) (Scheme 5).

In the first direction, for example, base-catalyzed arylation of **1a** by 2-naphthols occurs [44]. Direction B is essentially a new, unusual version of the synthetic realization of the C–C–dielectrophil + C–C–N–dinucleophil retrosynthetic scheme of the well-known method for the production of pyrroles by the Hantzsch reaction (interaction of α-halocarbonyl with β-enaminocarbonyl compounds [45,46]). The structural prerequisite for substantiating the possibility of using this approach for the synthesis of **8** is the high mobility of the bromine atom in bromomaleimides in reactions with nucleophilic reagents [47].

## 3. Materials and Methods

### 3.1. General

NMR ^1^Н and ^13^С spectra were registered on Bruker DRX (500 and 125.8 MHz, respectively) spectrometer in DMSO-*d*_6_, internal standard is TMS. Mass spectra were registered on Agilent Technologies LCMS 6230B (ESI, Agilent Technologies, Santa Clara, CA, USA). Melting points were determined on Stuart SMP 30. Control of reagent and product individuality, and qualitative analysis of reaction mass, was performed by TLC on Merck TLC Silicagel 60 F_254_ chromatographic plate (Merck KGaA, Darmstadt, Germany); eluents: methanol, chloroform, and their mixtures in various ratios. The chromatograms were developed by UV and iodine vapor.

Purity of the products was controlled by high performance liquid chromatography with high resolution mass-spectrometric detection under electrospray ionization (HPLC-HRMS-ESI) in combination with UV detection. The device consists of liquid chromatograph—Agilent 1269 Infinity and time-of-flight high resolution mass detector—Agilent 6230 TOF LC/MS (Agilent Technologies, Santa Clara, CA, USA). Block ionization is double electrospray, and the detection mass range is from 50 to 2000 Dalton. Capillary voltage is 4.0 kV, fragmentor +191 V, skimmer +66 V, OctRF 750 V. Column Poroshell 120 EC-C18 (4.6 × 50 mm; 2.7 mkm) was used. Gradient eluation: acetonitrile/water (0.1% formic acid); flow rate: 0.4 mL/min. Software for collection and elaboration of research results is MassHunter Workstation/Data Acquisition V.06.00. Starting bromomaleimides (**1**) and aminocrotonates (**2**) were provided by Alinda Chemical Ltd., Moscow, Russian Federation. Other reagents were purchased from commercial suppliers and used as received.

### 3.2. General Procedure for the Reaction of Bromomaleimides *(**1**)* with Aminocrotonates *(**3**)* and Characterization Data of Pyrrolo[3,4-b]pyrroles *(**8a**–**f**)*

*Two-component reaction*. A mixture of the corresponding bromomaleimide (0.002 mol) and aminocrotonate (0.004 mol) in 5 mL of methanol was stirred for 4 to 6 h. The precipitate which formed was filtered off and recrystallized from methanol.

*One-pot sequence*. A mixture of acetoacetic ester (**9**) (0.004 mol) and amine (**10**) (0.004 mol) in 3 mL of methanol was stirred for 24 h, after which, without isolation of the resulting aminocrotonate, a solution of the corresponding bromomaleimide (0.002 mol) in 5 mL of methanol was added. Stirring was continued for 4–6 h. The precipitate formed was filtered off and recrystallized from methanol. Hexahydropyrrolo[3,4-*b*]pyrrole (**8**) was obtained as colorless crystalline powders.

*(3aS,6aR)-Ethyl 1-benzyl-2-methyl-4,6-dioxo-5-phenyl-1,3a,4,5,6,6a-hexahydropyrrolo[3,4-b]pyrrole-3-carboxylate* **8a**. 0.36 g; yield 46%; m.p. 141–142 °С; ^1^H NMR (DMSO-*d*_6_), δ (ppm): 1.20 (3Н, t, *J* = 7.1 Hz, CH_3_CH_2_O); 2.31 (3Н, s, CH_3_-Het); 4.00–4.14 (2Н, m, CH_3_CH_2_O); 4.33 (1H, dd, *J* = 10.5 Hz, *J* = 0.8 Hz, H-6a); 4.53 (1H, d, *J* = 10.5 Hz, H-3a); 4.59 (1H, d, *J* = 16.7 Hz, CH_2_Ph); 4.80 (1H, d, *J* = 16.7 Hz, CH_2_Ph); 7.23–7.26 (4Н, m, CH_arom_); 7.30–7.34 (1Н, m, CH_arom_); 7.38–7.44 (3Н, m, CH_arom_); 7.47–7.51 (2Н, m, CH_arom_); ^13^C NMR (DMSO-*d*_6_), δ (ppm): 12.45, 14.90, 47.66, 48.16, 58.60, 63.24, 93.40, 127.37, 127.44, 127.86, 128.82, 129.21, 129.31, 132.63, 136.83, 161.46, 165.36, 173.83, 175.64; HRMS-ESI, *m*/*z* ([M + H]^+^), calcd for C_23_H_22_N_2_O_4_ + H^+^ 391.1654, found 391.1652.

*(3aS,6aR)-Ethyl 1-methoxybenzyl-2-methyl-4,6-dioxo-5-phenyl-1,3a,4,5,6,6a-hexahydropyrrolo[3,4-b]pyrrole-3-carboxylate* **8b**. 0.65 g; yield 77%; m.p. 152–153 °С; ^1^H NMR (DMSO-*d*_6_), δ (ppm): 1.20 (3Н, t, *J* = 7.1 Hz, CH_3_CH_2_O); 2.33 (3Н, s, CH_3_-Het); 3.75 (3Н, s, MeO); 3.99–4.13 (2Н, m, CH_3_CH_2_O); 4.29 (1H, br. d, *J* = 10.5 Hz, H-6a); 4.48 (1H, d, *J* = 16.2 Hz, CH_2_Ph); 4.49 (1H, d, *J* = 10.5 Hz, H-3a); 4.73 (1H, d, *J* = 16.2 Hz, CH_2_Ph); 6.96 (2H, d, *J* = 8.6 Hz, CH_arom_); 7.19 (2H, d, *J* = 8.6 Hz, CH_arom_); 7.23–7.26 (1Н, m, CH_arom_); 7.35–7.37 (1Н, m, CH_arom_); 7.40–7.44 (1Н, m, CH_arom_); 7.47–7.51 (2Н, m, CH_arom_); ^13^C NMR (DMSO-*d*_6_), δ (ppm): 12.48, 14.90, 47.59, 47.62, 55.48, 58.57, 62.88, 93.32, 114.60, 127.38, 128.32, 128.82, 129.07, 129.31, 132.63, 159.09, 161.35, 165.36, 173.86, 175.65; HRMS-ESI, *m*/*z* ([M + H]^+^), calcd for C_24_H_24_N_2_O_5_ + H^+^ 421.1759, found 421.1754.

*(3aS,6aR)-Ethyl 5-(4-ethylphenyl)-2-methyl-4,6-dioxo-1-phenetyl-1,3a,4,5,6,6a-hexahydropyrrolo[3,4-b]pyrrole-3-carboxylate* **8c**. 0.70 g; yield 82%; m.p. 147–149 °С; ^1^H NMR (DMSO-*d*_6_), δ (ppm): 1.18 (3Н, t, *J* = 7.1 Hz, 4-CH_3_CH_2_C_6_H_4_); 1.20 (3Н, t, *J* = 7.6 Hz, CH_3_CH_2_O); 2.07 (3Н, s, CH_3_-Het); 2.64 (2Н, q, *J* = 7.1 Hz, 4-CH_3_CH_2_C_6_H_4_); 2.80–2.87 (1Н, m, CH_2_CH_2_Ph); 2.92–2.99 (1Н, m, CH_2_CH_2_Ph); 3.60–3.70 (2Н, m, CH_2_CH_2_Ph); 3.96–4.10 (2Н, m, CH_3_CH_2_O); 4.28 (1H, dd, *J* = 10.5 Hz, *J* = 1.1 Hz, H-6a); 4.75 (1H, d, *J* = 10.5 Hz, H-3a); 7.14–7.17 (2Н, m, CH_arom_); 7.21–7.26 (3Н, m, CH_arom_); 7.30–7.34 (4Н, m, CH_arom_); ^13^C NMR (DMSO-*d*_6_), δ (ppm): 11.98, 14.90, 15.84, 28.21, 33.53, 46.93, 47.62, 58.43, 63.67, 92.92, 126.81, 127.25, 128.63, 128.88, 129.25, 130.24, 139.14, 144.54, 161.61, 165.30, 174.33, 175.75; HRMS-ESI, *m*/*z* ([M + H]^+^), calcd for C_26_H_28_N_2_O_4_+H^+^ 433.2123, found 433.2128.

*X-Ray Crystallographic data for* **8c**. CCDC 1574386 contains the supplementary crystallographic data for this paper. These data can be obtained free of charge via http://www.ccdc.cam.ac.uk/conts/retrieving.html (or from the CCDC, 12 Union Road, Cambridge CB2 1EZ, UK; Fax: +44-1223-336033; E-mail: deposit@ccdc.cam.ac.uk). Crystal data for C_26_H_28_N_2_O_4_ (M = 432.50 g/mol): monoclinic, space group P 21/n (no. 14), a = 15.0252(9) Å, b = 8.6768(5) Å, c = 17.3436(10) Å, α = 90°, β = 97.7760(10)°, γ = 90° V = 2240.3(2) Å3, Z = 4, T =120(2) K, μ(CuKα) = 0.087 mm-1, Dcalc = 1.282 g/cm^3^. 20916 reflections measured (4.74° ≤ 2Θ ≤ 51.992°), 4387 unique (Rint = 0.0380, Rsigma = 0.0411) which were used in all calculations. The final R1 was 0.0483 (I > 2σ(I)) and wR2 was 0.0817 (all data).

*(3aS,6aR)-Ethyl 5-(4-ethoxyphenyl)-2-methyl-4,6-dioxo-1-phenetyl-1,3a,4,5,6,6a-hexahydropyrrolo[3,4-b]pyrrole-3-carboxylate* **8d**. 0.66 g; yield 73%; m.p. 164–166 °С; ^1^H NMR (DMSO-*d*_6_), δ (ppm): 1.20 (3Н, t, *J* = 7.0 Hz, CH_3_CH_2_O); 1.34 (3Н, t, *J* = 7.0 Hz, 4-CH_3_CH_2_OC_6_H_4_); 2.06 (3Н, s, CH3-Het); 2.78–2.87 (1Н, m, CH_2_CH_2_Ph); 2.91–2.96 (1Н, m, CH_2_CH_2_Ph); 3.60–3.69 (2Н, m, CH_2_CH_2_Ph); 4.04–4.12 (4Н, m, 2CH_3_CH_2_); 4.27 (1H, br.d, *J* = 10.5 Hz, H-6a); 4.74 (1H, d, *J* = 10.5 Hz, H-3a); 7.01 (2H, d, *J* = 8.7 Hz, CH_arom_); 7.15 (2H, d, *J* = 8.7 Hz, CH_arom_); 7.23–7.26 (3Н, m, CH_arom_); 7.30–7.33 (2Н, m, CH_arom_); ^13^C NMR (DMSO-*d*_6_), δ (ppm): 11.97, 14.91, 14.96, 33.53, 46.91, 47.54, 58.42, 63.59, 63.70, 92.91, 114.96, 124.99, 126.81, 128.58, 128.88, 129.25, 139.15, 158.63, 161.58, 165.31, 174.42, 175.86; HRMS-ESI, *m*/*z* ([M + H]^+^), calcd. for C_26_H_28_N_2_O_5_ + H^+^ 449.2073, found 449.2067.

*(3aS,6aR)-Ethyl 1-benzyl-2-methyl-4,6-dioxo-5-(3,4-dichlorophenyl)-1,3a,4,5,6,6a-hexahydropyrrolo[3,4-b]pyrrole-3-carboxylate* **8e***.* 0.64 g; yield 70%; m.p. 146–147 °С; ^1^H NMR (DMSO-*d*_6_), δ (ppm): 1.20 (3Н, t, *J* = 7.1 Hz, CH_3_CH_2_O); 2.31 (3Н, s, CH_3_-Het); 4.00–4.14 (2Н, m, CH_3_CH_2_O); 4.33 (1H, br.d, *J* = 10.5 Hz, H-6a); 4.52 (1H, d, *J* = 10.5 Hz, H-3a); 4.59 (1H, d, *J* = 16.7 Hz, CH_2_Ph); 4.78 (1H, d, *J* = 16.7 Hz, CH_2_Ph); 7.25 (2H, d, *J* = 7.4 Hz, CH_arom_); 7.30–7.34 (2Н, m, CH_arom_); 7.40 (2H, t, *J* = 7.5 Hz, CH_arom_); 7.61 (1H, d, *J* = 2.3 Hz, CH_arom_); 7.79 (1H, d, *J* = 8.6 Hz, CH_arom_); ^13^C NMR (DMSO-*d*_6_), δ (ppm): 12.48, 14.90, 47.74, 48.12, 58.60, 63.15, 93.12, 127.49, 127.75, 127.87, 129.19, 129.27, 131.30, 131.53, 131.61, 132.52, 136.76, 161.61, 165.29, 173.27, 175.11; HRMS-ESI, *m*/*z* ([M + H]^+^), calcd for C_23_H_20_Cl_2_N_2_O_4_ + H^+^ 459.0874, found 459.0868.

*(3aS,6aR)-Ethyl 5-(3,4-dichlorophenyl)-2-methyl-4,6-dioxo-1-phenetyl-1,3a,4,5,6,6a-hexahydropyrrolo[3,4-b]pyrrole-3-carboxylate* **8f***.* 0.65 g; yield 69%; m.p. 155–156 °С; ^1^H NMR (DMSO-*d*_6_), δ (ppm): 1.18 (3Н, t, *J* = 7.1 Hz, CH_3_CH_2_O); 2.08 (3Н, s, CH_3_-Het); 2.81–2.88 (1Н, m, CH_2_CH_2_Ph); 2.93–2.99 (1Н, m, CH_2_CH_2_Ph); 3.60–3.67 (2Н, m, CH_2_CH_2_Ph); 3.98–4.10 (2Н, m, CH_3_CH_2_); 4.28 (1H, dd, *J* = 10.6 Hz, *J* = 1.0 Hz, H-6a); 4.75 (1H, d, *J* = 10.6 Hz, H-3a); 7.22–7.27 (3Н, m, CH_arom_); 7.30–7.35 (3Н, m, CH_arom_); 7.64 (1H, d, *J* = 2.3 Hz, CH_arom_); 7.79 (1H, d, *J* = 8.6 Hz, CH_arom_); ^13^C NMR (DMSO-*d*_6_), δ (ppm): 12.05, 14.91, 14.96, 33.48, 46.81, 47.74, 58.45, 63.56, 92.63, 126.82, 127.83, 128.88, 129.01, 129.25, 129.32, 131.31, 131.53, 131.60, 132.58, 139.14, 161.80, 165.23, 173.69, 175.14; HRMS, *m*/*z* ([M + H]^+^), calcd for C_24_H_22_Cl_2_N_2_O_4_ + H^+^ 473.1031, found 473.1024.

## 4. Conclusions

Herein, we presented the new unusual variant of the realization of the Hantzsch-type synthetic scheme C–C + C–C–N for the synthesis of polyhydrogenated pyrrolo[3,4-*b*]pyrroles based on the cyclization of bromomaleimides with aminocrotonic acid esters. A domino-reaction proceeds chemo- and stereoselectively and involves the steps of intermolecular nucleophilic C-addition or substitution and intramolecular nucleophilic N-addition both in two- and multicomponent mode.

## Figures and Tables

**Table 1:** Reaction of **1** with **2**.

Entry	Bromomaleimide, Ar	Aminocrotonate, R	Product	Time (h)	Yields ^1^ (%)
1	Ph (**1a**)	Ph (**2a**)	**8a**	5	46/53
2	Ph (**1a**)	4-MeOC_6_H_4_ (**2b**)	**8b**	5	77/69
3	4-EtC_6_H_4_ (**1b**)	PhCH_2_ (**2c**)	**8c**	4	82/74
4	4-EtOC_6_H_4_ (**1c**)	PhCH_2_ (**2c**)	**8d**	6	73/70
5	3,4-Cl_2_C_6_H_3_ (**1d**)	Ph (**2a**)	**8e**	6	70/64
6	3,4-Cl_2_C_6_H_3_ (**1d**)	PhCH_2_ (**2c**)	**8f**	6	69/61

^1^ Isolated yields; in two component reaction/in one-pot synthesis.

## Supplementary Materials

The NMR spectra, data of HPLC-MS-ESI analysis of pyrrolopyrroles **8** and crystallographic data for 8c are available online.
