# Supplementary material for: A New Synthetic Route to Polyhydrogenated Pyrrolo[3,4-b]pyrroles by the Domino Reaction of 3-Bromopyrrole-2,5-Diones with Aminocrotonic Acid Esters
Source: Molecules. 2017 Nov 22;22(11):2035. doi: 10.3390/molecules22112035 (PMC6150208; doi:10.3390/molecules22112035)
Supplement: Supplementary file 1 [file molecules-22-02035-s001.zip › Supplement material.doc]

**Supplementary Materials:**

**A new synthetic route to polyhydrogenated pyrrolo[3,4-*b*]pyrroles by the domino reaction of 3-bromopyrrole-2,5-diones with aminocrotonic acid esters**

Khidmet Shikhaliev, Artem Sabynin, Valeri Sekirin, Michael Krysin, Fedor Zubkov^,^ and Kristina Yankina

**^1^H, ^13^C NMR spectra and data of HPLC-MS-ESI analysis of pyrrolopyrroles 8**

**(3a*S*,6a*R*)-Ethyl 1-benzyl-2-methyl-4,6-dioxo-5-phenyl-1,3a,4,5,6,6a-hexahydropyrrolo[3,4-*b*]pyrrole-3-carboxylate 8a**

**(3a*S*,6a*R*)-Ethyl 1-methoxybenzyl-2-methyl-4,6-dioxo-5-phenyl-1,3a,4,5,6,6a-hexahydropyrrolo[3,4-*b*]pyrrole-3-carboxylate** **8b**

**(3a*S*,6a*R*)-Ethyl 5-(4-ethylphenyl)-2-methyl-4,6-dioxo-1-phenetyl-1,3a,4,5,6,6a-hexahydropyrrolo[3,4-*b*]pyrrole-3-carboxylate 8c**

**(3a*S*,6a*R*)-Ethyl 5-(4-ethoxyphenyl)-2-methyl-4,6-dioxo-1-phenetyl-1,3a,4,5,6,6a-hexahydropyrrolo[3,4-*b*]pyrrole-3-carboxylate** **8d**

**(3a*S*,6a*R*)-Ethyl 1-benzyl-2-methyl-4,6-dioxo-5-(3,4-dichlorophenyl)-1,3a,4,5,6,6a-hexahydropyrrolo[3,4-*b*]pyrrole-3-carboxylate** **8e**

**(3a*S*,6a*R*)-Ethyl 5-(3,4-dichlorophenyl)-2-methyl-4,6-dioxo-1-phenetyl-1,3a,4,5,6,6a-hexahydropyrrolo[3,4-*b*]pyrrole-3-carboxylate 8f**

**X-Ray crystallographic data for (3a*S*,6a*R*)-ethyl 5-(4-ethylphenyl)-2-methyl-4,6-dioxo-1-phenetyl-1,3a,4,5,6,6a-hexahydropyrrolo[3,4-*b*]pyrrole-3-carboxylate** **8c.**

CCDC 1574386 contains the supplementary crystallographic data for this paper. These data can be obtained free of charge via http://www.ccdc.cam.ac.uk/conts/retrieving.html (or from the CCDC, 12 Union Road, Cambridge CB2 1EZ, UK; Fax: +44 1223 336033; E-mail: [deposit@ccdc.cam.ac.uk](mailto:deposit@ccdc.cam.ac.uk)). Crystal data for C_26_H_28_N_2_O_4_ (M = 432.50 g/mol): monoclinic, space group P 21/n (no. [14](http://it.iucr.org/cgi-bin/gotosgtable.pl?number=14&tabletype=S)), a = 15.0252(9) Å, b = 8.6768(5) Å, c = 17.3436(10) Å, α = 90°, β = 97.7760(10)°, γ = 90° V = 2240.3(2) Å3, Z = 4, T =120(2) K, μ(CuKα) = 0.087 mm-1, Dcalc = 1.282 g/cm^3^. 20916 reflections measured (4.74° ≤ 2Θ ≤ 51.992°), 4387 unique (Rint = 0.0380, Rsigma = 0.0411) which were used in all calculations. The final R1 was 0.0483 (I > 2σ(I)) and wR2 was 0.0817 (all data).

**Figure 1.** The general form of the molecule **8c** in the crystal from X-ray diffraction studies. Non-hydrogen atoms are represented by probabilistic ellipsoids of atomic displacements (p = 0.5).

**Figure 2.** Fragment of the crystalline package **8c** along the crystallographic axis b.

An X-ray diffraction study of compound **8c** was carried out on an APEX II CCD diffractometer (MoKα radiation, graphite monochromator, ω scan). The structure is deciphered by a direct method and refined by the least squares in the anisotropic full-matrix approximation by F2hkl. The positions of all hydrogen atoms are calculated from geometric considerations. All hydrogen atoms are refined in the isotropic approximation in the rider model. All calculations were carried out using the SHELXTL PLUS program complex [1].

**References**

1. Sheldrick, G.M. A short history of SHELX. *Acta Cryst*. **2008**, *A64*, 112-122, <https://doi.org/10.1107/S0108767307043930>

Table 1. Crystal data and structure refinement for **8с**.

Identification code	x

Empirical formula	C26 H28 N2 O4

Formula weight	432.50

Temperature	120(2) K

Wavelength	0.71073 Å

Crystal system	Monoclinic

Space group	P2**_1_**/n

Unit cell dimensions	a = 15.0252(9) Å	α= 90°.

b = 8.6768(5) Å	β= 97.7760(10)°.

c = 17.3436(10) Å	γ = 90°.

Volume	2240.3(2) Å^3^

Z	4

Density (calculated)	1.282 Mg/m^3^

Absorption coefficient	0.087 mm^-1^

F(000)	920

Crystal size	0.270 x 0.210 x 0.160 mm^3^

Theta range for data collection	2.370 to 25.996°.

Index ranges	-18<=h<=18, -10<=k<=10, -21<=l<=21

Reflections collected	20916

Independent reflections	4387 [R(int) = 0.0380]

Completeness to theta = 25.242°	99.3 %

Absorption correction	Semi-empirical from equivalents

Max. and min. transmission	0.995 and 0.962

Refinement method	Full-matrix least-squares on F^2^

Data / restraints / parameters	4387 / 0 / 293

Goodness-of-fit on F^2^	1.335

Final R indices [I>2sigma(I)]	R1 = 0.0483, wR2 = 0.0817

R indices (all data)	R1 = 0.0704, wR2 = 0.0879

Extinction coefficient	n/a

Largest diff. peak and hole	0.794 and -0.338 e. Å^-3^

Table 2. Atomic coordinates ( x 10^4^) and equivalent isotropic displacement parameters (Å^2^x 10^3^)

for **8с**. U(eq) is defined as one third of the trace of the orthogonalized Uij tensor.

________________________________________________________________________________

x	y	z	U(eq)

________________________________________________________________________________

O(15)	8248(1)	9871(2)	1671(1)	38(1)

O(17)	6242(1)	9111(2)	1846(1)	30(1)

O(27)	10021(1)	8704(2)	3940(1)	33(1)

O(30)	5706(1)	10990(2)	2555(1)	34(1)

N(1)	9290(1)	9531(2)	2757(1)	23(1)

N(6)	8044(1)	9508(2)	4134(1)	26(1)

C(2)	8432(1)	9401(2)	2324(1)	25(1)

C(3)	7813(1)	8615(2)	2829(1)	23(1)

C(4)	7074(1)	9678(2)	3019(1)	24(1)

C(5)	7277(1)	10172(2)	3771(1)	25(1)

C(7)	8400(1)	8389(2)	3618(1)	25(1)

C(8)	9344(1)	8849(2)	3484(1)	25(1)

C(9)	10060(1)	10113(2)	2436(1)	24(1)

C(10)	10761(2)	9150(3)	2356(2)	49(1)

C(11)	11497(2)	9701(3)	2039(2)	56(1)

C(12)	11545(1)	11208(2)	1798(1)	36(1)

C(13)	10838(1)	12155(3)	1903(1)	46(1)

C(14)	10095(1)	11624(2)	2216(1)	41(1)

C(16)	6286(1)	10028(2)	2482(1)	26(1)

C(18)	6765(1)	11291(2)	4191(1)	34(1)

C(19)	8416(1)	9642(2)	4952(1)	29(1)

C(20)	8222(1)	8249(2)	5451(1)	32(1)

C(21)	7233(1)	7929(2)	5426(1)	31(1)

C(22)	6806(1)	6881(2)	4897(1)	35(1)

C(23)	5890(1)	6638(3)	4839(1)	44(1)

C(24)	5386(2)	7448(3)	5309(2)	52(1)

C(25)	5805(2)	8488(3)	5846(2)	54(1)

C(26)	6720(2)	8719(2)	5906(1)	42(1)

C(31)	5471(1)	9302(2)	1258(1)	34(1)

C(32)	5639(2)	8325(3)	587(1)	48(1)

C(27)	12324(2)	11814(3)	1404(2)	53(1)

C(28)	13173(2)	10843(3)	1521(2)	56(1)

Table 3. Bond lengths [Å] and angles [°] for **8с**.

_____________________________________________________

O(15)-C(2)	1.200(2)

O(17)-C(16)	1.355(2)

O(17)-C(31)	1.445(2)

O(27)-C(8)	1.208(2)

O(30)-C(16)	1.225(2)

N(1)-C(8)	1.384(2)

N(1)-C(2)	1.407(2)

N(1)-C(9)	1.441(2)

N(6)-C(5)	1.364(2)

N(6)-C(19)	1.458(2)

N(6)-C(7)	1.470(2)

C(2)-C(3)	1.523(2)

C(3)-C(4)	1.513(2)

C(3)-C(7)	1.536(2)

C(4)-C(5)	1.368(3)

C(4)-C(16)	1.437(2)

C(5)-C(18)	1.488(3)

C(7)-C(8)	1.521(2)

C(9)-C(10)	1.367(3)

C(9)-C(14)	1.368(3)

C(10)-C(11)	1.384(3)

C(11)-C(12)	1.378(3)

C(12)-C(13)	1.373(3)

C(12)-C(27)	1.528(3)

C(13)-C(14)	1.385(3)

C(19)-C(20)	1.536(3)

C(20)-C(21)	1.506(3)

C(21)-C(22)	1.386(3)

C(21)-C(26)	1.389(3)

C(22)-C(23)	1.382(3)

C(23)-C(24)	1.379(3)

C(24)-C(25)	1.385(3)

C(25)-C(26)	1.380(3)

C(31)-C(32)	1.489(3)

C(27)-C(28)	1.518(3)

C(16)-O(17)-C(31)	116.96(14)

C(8)-N(1)-C(2)	112.90(15)

C(8)-N(1)-C(9)	123.38(14)

C(2)-N(1)-C(9)	123.15(15)

C(5)-N(6)-C(19)	127.17(16)

C(5)-N(6)-C(7)	110.62(14)

C(19)-N(6)-C(7)	121.53(15)

O(15)-C(2)-N(1)	123.70(17)

O(15)-C(2)-C(3)	127.94(17)

N(1)-C(2)-C(3)	108.35(15)

C(4)-C(3)-C(2)	112.03(15)

C(4)-C(3)-C(7)	103.52(14)

C(2)-C(3)-C(7)	104.36(14)

C(5)-C(4)-C(16)	127.51(17)

C(5)-C(4)-C(3)	108.87(15)

C(16)-C(4)-C(3)	123.58(16)

N(6)-C(5)-C(4)	111.90(16)

N(6)-C(5)-C(18)	120.68(16)

C(4)-C(5)-C(18)	127.42(17)

N(6)-C(7)-C(8)	110.11(15)

N(6)-C(7)-C(3)	104.23(14)

C(8)-C(7)-C(3)	105.40(14)

O(27)-C(8)-N(1)	125.57(17)

O(27)-C(8)-C(7)	126.33(17)

N(1)-C(8)-C(7)	108.05(14)

C(10)-C(9)-C(14)	119.87(18)

C(10)-C(9)-N(1)	119.67(17)

C(14)-C(9)-N(1)	120.45(17)

C(9)-C(10)-C(11)	119.8(2)

C(12)-C(11)-C(10)	121.7(2)

C(13)-C(12)-C(11)	117.03(19)

C(13)-C(12)-C(27)	120.4(2)

C(11)-C(12)-C(27)	122.50(19)

C(12)-C(13)-C(14)	122.1(2)

Table 4. Anisotropic displacement parameters (Е^2^x 10^3^) for **8с**. The anisotropic

displacement factor exponent takes the form: -2π^2^[ h^2^ a*^2^U^11^ + ... + 2 h k a* b* U^12^ ]

______________________________________________________________________________

U^11^	U^22^	U^33^	U^23^	U^13^	U^12^

______________________________________________________________________________

O(15)	32(1)	52(1)	27(1)	11(1)	-2(1)	0(1)

O(17)	24(1)	30(1)	32(1)	-6(1)	-7(1)	3(1)

O(27)	24(1)	46(1)	28(1)	4(1)	-4(1)	1(1)

O(30)	29(1)	31(1)	40(1)	-4(1)	-4(1)	7(1)

N(1)	22(1)	24(1)	24(1)	2(1)	1(1)	0(1)

N(6)	25(1)	30(1)	23(1)	1(1)	1(1)	0(1)

C(2)	25(1)	25(1)	24(1)	0(1)	0(1)	3(1)

C(3)	21(1)	21(1)	26(1)	1(1)	-2(1)	-1(1)

C(4)	22(1)	20(1)	28(1)	0(1)	1(1)	-1(1)

C(5)	22(1)	23(1)	30(1)	2(1)	4(1)	-3(1)

C(7)	25(1)	24(1)	26(1)	3(1)	1(1)	0(1)

C(8)	25(1)	24(1)	26(1)	-1(1)	1(1)	1(1)

C(9)	23(1)	24(1)	25(1)	-1(1)	2(1)	-1(1)

C(10)	51(1)	26(1)	76(2)	14(1)	34(1)	8(1)

C(11)	48(1)	40(1)	88(2)	14(1)	38(1)	16(1)

C(12)	31(1)	35(1)	43(1)	4(1)	9(1)	0(1)

C(13)	39(1)	25(1)	77(2)	9(1)	18(1)	-2(1)

C(14)	31(1)	26(1)	68(2)	4(1)	16(1)	6(1)

C(16)	23(1)	22(1)	31(1)	0(1)	1(1)	-3(1)

C(18)	32(1)	37(1)	32(1)	-4(1)	4(1)	4(1)

C(19)	28(1)	34(1)	24(1)	1(1)	1(1)	-4(1)

C(20)	33(1)	36(1)	25(1)	4(1)	1(1)	-3(1)

C(21)	34(1)	30(1)	29(1)	3(1)	7(1)	-3(1)

C(22)	35(1)	37(1)	34(1)	-6(1)	11(1)	-2(1)

C(23)	37(1)	46(1)	49(1)	-14(1)	9(1)	-9(1)

C(24)	34(1)	53(2)	74(2)	-16(1)	17(1)	-7(1)

C(25)	49(1)	47(2)	72(2)	-22(1)	31(1)	-7(1)

C(26)	46(1)	36(1)	47(1)	-14(1)	17(1)	-12(1)

C(31)	25(1)	35(1)	37(1)	-3(1)	-9(1)	1(1)

C(32)	44(1)	56(2)	39(1)	-13(1)	-12(1)	10(1)

C(27)	36(1)	55(2)	72(2)	16(1)	18(1)	-2(1)

C(28)	36(1)	53(2)	84(2)	-20(1)	28(1)	-10(1)

Table 5. Hydrogen coordinates ( x 10^4^) and isotropic displacement parameters (Е^2^x 10 ^3^)

for **8с**.

________________________________________________________________________________

x	y	z	U(eq)

________________________________________________________________________________

H(3A)	7571	7617	2600	28

H(7A)	8374	7309	3813	30

H(10A)	10744	8106	2518	59

H(11A)	11981	9022	1987	67

H(13A)	10861	13207	1757	55

H(14A)	9613	12303	2278	49

H(18A)	6221	11609	3851	50

H(18B)	7139	12196	4337	50

H(18C)	6596	10804	4660	50

H(19A)	8166	10578	5170	35

H(19B)	9074	9778	4990	35

H(20A)	8517	7327	5265	38

H(20B)	8487	8438	5996	38

H(22A)	7148	6321	4569	42

H(23A)	5608	5913	4474	53

H(24A)	4755	7293	5266	63

H(25A)	5461	9044	6173	65

H(26A)	7003	9426	6279	51

H(31A)	5403	10396	1099	40

H(31B)	4916	8968	1460	40

H(32A)	5129	8409	173	72

H(32B)	5711	7249	755	72

H(32C)	6187	8675	392	72

H(27A)	12120	11900	838	64

H(27B)	12476	12865	1602	64

H(28A)	13652	11379	1298	84

H(28B)	13056	9846	1261	84

H(28C)	13357	10678	2078	84

________________________________________________________________________________

Table 6. Torsion angles [°] for **8с**.

________________________________________________________________

C(8)-N(1)-C(2)-O(15)	-177.61(18)

C(9)-N(1)-C(2)-O(15)	-6.0(3)

C(8)-N(1)-C(2)-C(3)	3.4(2)

C(9)-N(1)-C(2)-C(3)	175.02(15)

O(15)-C(2)-C(3)-C(4)	-64.5(3)

N(1)-C(2)-C(3)-C(4)	114.37(16)

O(15)-C(2)-C(3)-C(7)	-175.87(19)

N(1)-C(2)-C(3)-C(7)	3.02(19)

C(2)-C(3)-C(4)-C(5)	-104.09(17)

C(7)-C(3)-C(4)-C(5)	7.78(19)

C(2)-C(3)-C(4)-C(16)	78.0(2)

C(7)-C(3)-C(4)-C(16)	-170.12(16)

C(19)-N(6)-C(5)-C(4)	-173.61(17)

C(7)-N(6)-C(5)-C(4)	-3.1(2)

C(19)-N(6)-C(5)-C(18)	6.4(3)

C(7)-N(6)-C(5)-C(18)	177.00(16)

C(16)-C(4)-C(5)-N(6)	174.52(17)

C(3)-C(4)-C(5)-N(6)	-3.3(2)

C(16)-C(4)-C(5)-C(18)	-5.5(3)

C(3)-C(4)-C(5)-C(18)	176.67(17)

C(5)-N(6)-C(7)-C(8)	120.41(16)

C(19)-N(6)-C(7)-C(8)	-68.4(2)

C(5)-N(6)-C(7)-C(3)	7.79(19)

C(19)-N(6)-C(7)-C(3)	178.97(15)

C(4)-C(3)-C(7)-N(6)	-9.05(17)

C(2)-C(3)-C(7)-N(6)	108.33(15)

C(4)-C(3)-C(7)-C(8)	-125.01(15)

C(2)-C(3)-C(7)-C(8)	-7.63(18)

C(2)-N(1)-C(8)-O(27)	174.08(18)

C(9)-N(1)-C(8)-O(27)	2.5(3)

C(2)-N(1)-C(8)-C(7)	-8.5(2)

C(9)-N(1)-C(8)-C(7)	179.90(15)

N(6)-C(7)-C(8)-O(27)	75.4(2)

C(3)-C(7)-C(8)-O(27)	-172.71(18)

N(6)-C(7)-C(8)-N(1)	-101.92(16)

C(3)-C(7)-C(8)-N(1)	9.94(19)

C(8)-N(1)-C(9)-C(10)	57.2(3)

C(2)-N(1)-C(9)-C(10)	-113.5(2)

C(8)-N(1)-C(9)-C(14)	-122.3(2)

C(2)-N(1)-C(9)-C(14)	67.0(3)

C(14)-C(9)-C(10)-C(11)	-1.2(4)

N(1)-C(9)-C(10)-C(11)	179.2(2)

C(9)-C(10)-C(11)-C(12)	-0.1(4)

C(10)-C(11)-C(12)-C(13)	1.6(4)

C(10)-C(11)-C(12)-C(27)	-176.4(2)

C(11)-C(12)-C(13)-C(14)	-1.8(4)

C(27)-C(12)-C(13)-C(14)	176.1(2)

C(10)-C(9)-C(14)-C(13)	1.0(3)

N(1)-C(9)-C(14)-C(13)	-179.49(19)

C(12)-C(13)-C(14)-C(9)	0.6(4)

C(31)-O(17)-C(16)-O(30)	-0.8(3)

C(31)-O(17)-C(16)-C(4)	178.64(16)

C(5)-C(4)-C(16)-O(30)	10.9(3)

C(3)-C(4)-C(16)-O(30)	-171.64(18)

C(5)-C(4)-C(16)-O(17)	-168.54(17)

C(3)-C(4)-C(16)-O(17)	9.0(2)

C(5)-N(6)-C(19)-C(20)	100.5(2)

C(7)-N(6)-C(19)-C(20)	-69.1(2)

N(6)-C(19)-C(20)-C(21)	-57.8(2)

C(19)-C(20)-C(21)-C(22)	93.7(2)

C(19)-C(20)-C(21)-C(26)	-83.4(2)

C(26)-C(21)-C(22)-C(23)	0.8(3)

C(20)-C(21)-C(22)-C(23)	-176.4(2)

C(21)-C(22)-C(23)-C(24)	0.2(4)

C(22)-C(23)-C(24)-C(25)	-0.8(4)

C(23)-C(24)-C(25)-C(26)	0.4(4)

C(24)-C(25)-C(26)-C(21)	0.7(4)

C(22)-C(21)-C(26)-C(25)	-1.3(3)

C(20)-C(21)-C(26)-C(25)	175.9(2)

C(16)-O(17)-C(31)-C(32)	172.89(17)

C(13)-C(12)-C(27)-C(28)	163.4(2)

C(11)-C(12)-C(27)-C(28)	-18.7(4)
